# A Study on the Iodine Vapor Adsorption Performance and Desorption Behavior of HKUST-1 with Varying Particle Sizes

**DOI:** 10.3390/molecules30030502

**Published:** 2025-01-23

**Authors:** Tian Lan, Xiaofan Ding, Qi Chen, Songtao Xiao, Taihong Yan, Tianchi Li, Weifang Zheng

**Affiliations:** China Institute of Atomic Energy, P.O. Box 275 (26), Beijing 102413, China; lant3@163.com (T.L.); dxf1102127620@126.com (X.D.); larychen1995@163.com (Q.C.); xiao_songtao@126.com (S.X.); yanthcn@163.com (T.Y.)

**Keywords:** spent fuel reprocessing, oxidative volatilization, ^129^I, MOFs, adsorption capacity

## Abstract

Iodine is one of the key elements that must be removed from the off-gas systems of nuclear fuel reprocessing. This study systematically investigates the iodine vapor adsorption performance of the metal–organic framework (MOF) material HKUST-1(1-(2-methyl-4-(2-oxopyrrolidin-1-yl)phenyl)-3-morpholino-5,6-dihydropyridin-2(1H)-one), with particle sizes of 100 nm and 20 μm. HKUST-1 samples with varying particle sizes were synthesized via a hydrothermal method. The experimental results show that the 20 μm HKUST-1 exhibits superior crystallinity, a more intact pore structure, and a higher iodine adsorption capacity, reaching 700 mg/g, which is significantly greater than the 300 mg/g capacity of the 100 nm HKUST-1. Kinetic analysis reveals that the adsorption process follows the pseudo-second-order model, with physical adsorption as the predominant mechanism, where iodine molecules are accommodated within the pores. FTIR and XRD further confirm the structural stability of the HKUST-1 framework after iodine adsorption. However, desorption experiments show that iodine molecules are easily volatilized into the air, with a 20% weight loss observed within 10 h and a color change from black to green. The results provide experimental evidence for optimizing the application of HKUST-1 materials in iodine capture and suggest that material modification could enhance the long-term stability of iodine fixation.

## 1. Introduction

Iodine is a significant fission product in reactor spent fuel, with several isotopes. Among them, ^129^I stands out due to its long half-life (1.57 × 10^7^ years), remaining in spent fuel even after many years of cooling. In spent fuel with a burnup of 3.3 GW/dtU, approximately 270 g/tU (1.388 × 10^9^ Bq/tU) of ^129^I is present, and its concentration increases with higher burnup levels [1]. Iodine is released during processes such as shearing, high-temperature oxidative volatilization, and dissolution of spent fuel, with the majority of iodine entering the off-gas stream [2]. Due to the strong radioactivity of ^129^I, its long half-life, and its high environmental mobility, iodine released into the atmosphere can migrate to living organisms or the human body via radionuclide transfer, posing significant health risks [3]. Therefore, it is crucial to capture iodine from the off-gas and ultimately stabilize it in a form that allows for long-term geological storage [4,5,6].

Currently, iodine capture and adsorption methods primarily include liquid scrubbing techniques such as the Mercurex process, Iodex process, electrochemical washing, and caustic alkali washing, as well as solid adsorption methods using metal-exchanged zeolites, silver-modified colloids, and porous organic compounds. However, these methods have various drawbacks. The Mercurex process uses large quantities of highly toxic and corrosive mercury, making it cumbersome to handle [7,8]. The Iodex process employs 20–23 mol/L nitric acid, which causes severe corrosion to equipment [7]. Electrochemical methods generate byproducts like Co(NO_3_)_2_ and iodates, which are unsuitable for storage [9,10]. The caustic alkali washing method produces large volumes of radioactive wastewater and is inefficient in removing organic iodine [7].

Solid capture methods involve adsorbing radioactive iodine onto high-surface-area materials, with activated carbon and zeolites being commonly used. While activated carbon effectively adsorbs iodine, its adsorption is primarily physical, and iodine can easily desorb under high-temperature conditions [11]. Zeolites, however, require modification, such as silver loading (e.g., AgX or AgZ), to adsorb iodine via a chemical reaction that forms AgI, which is stable at high temperatures and has low solubility in water [12,13,14,15,16,17,18]. However, the high temperatures required to convert iodine-loaded zeolites into stable waste forms present challenges [19]. Moreover, the high cost of silver limits its widespread use.

Metal–organic frameworks (MOFs) have recently emerged as a promising class of materials for adsorption applications. Due to their zeolite-like structures, tunable pores, and large surface areas, MOFs have garnered significant research interest. HKUST-1, first synthesized in 1991 by the Williams group at the Hong Kong University of Science and Technology, is one of the most well-known MOFs. Also known as Gu-BTC, HKUST-1 consists of copper(II) clusters and benzene-1,3,5-tricarboxylate ligands, forming a three-dimensional open-pore structure with a pore diameter of approximately 0.9 nm, a pore volume of 0.7 cm^3^/g, and a surface area of about 1798 m^2^/g. It shows good stability below 280 °C and excellent adsorption capacities for gasses such as CO_2_, H_2_, alkanes, and aromatic hydrocarbons [20,21,22,23].

Dorina F. Sava and colleagues studied the competitive adsorption of iodine and water on HKUST-1 [24], finding that iodine is preferentially adsorbed in the pores, inhibiting water adsorption. The iodine adsorption capacity of HKUST-1 can reach up to 175 wt%. However, the long-term iodine adsorption performance and the stability of iodine-loaded HKUST-1 have not been thoroughly examined. To further understand the iodine adsorption properties of HKUST-1, this study used I-127 instead of I-129 for the experiments and investigated the long-term iodine adsorption performance of HKUST-1 with different particle sizes.

## 2. Results and Discussion

### 2.1. Characterization of HKUST-1

**Figure 1:**
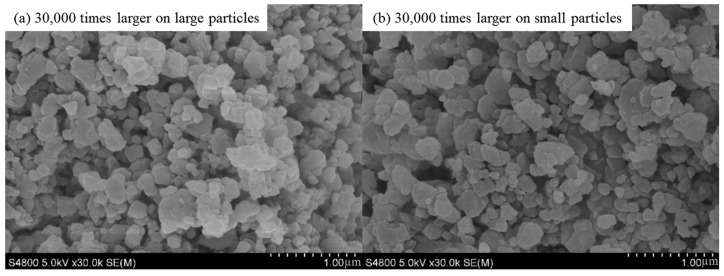
SEM image of 100 nm HKUST-1 (**a**) 30,000 times larger on large particles (**b**) 30,000 times larger on small particles.

**Figure 2:**
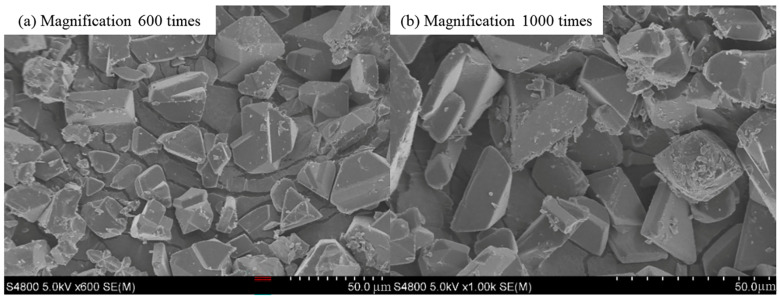
SEM image of 20 μm HKUST-1 (**a**) magnification 600 times (**b**) magnification 1000 times.

The morphology of the synthesized HKUST-1 crystals was characterized using scanning electron microscopy (SEM), and the results are shown in Figure 1 and Figure 2. As seen in Figure 1, the synthesized HKUST-1 crystals primarily exhibit particle sizes ranging from 80 nm to 120 nm, with a relatively regular shape and uniform size distribution. The formation of these nano-sized particles is mainly attributed to the mild conditions and uniform nucleation mechanism of the hydrothermal synthesis process. The smaller particle size and uniform distribution help increase the material’s specific surface area and enhance the exposure of its pore structure, thereby improving the iodine adsorption performance of HKUST-1. Additionally, the uniform particle size minimizes issues such as pore blockage and uneven diffusion during the experiment, facilitating the adsorption kinetics. Figure 2 shows that the particle size distribution of the 20 μm HKUST-1 crystals ranges from 5 μm to 30 μm, with most crystals being approximately 20 μm in size. The crystal morphology exhibits a typical cuboid-like structure. Compared to the 100 nm HKUST-1, the 20 μm crystals are larger, with smoother surfaces and well-defined edges. This suggests that during synthesis, the lower nucleation density and higher crystal growth rate resulted in crystals with higher crystallinity.

**Figure 3:**
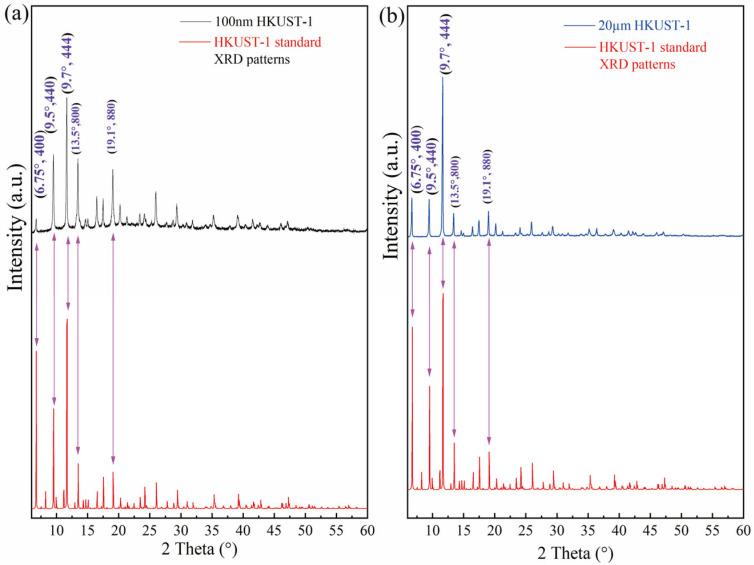
XRD patterns of (**a**) 100 nm and (**b**) 20 μm HKUST-1.

Figure 3a,b show the X-ray diffraction (XRD) patterns of the synthesized 100 nm and 20 μm HKUST-1 samples, aimed at verifying the crystal structure and crystallinity of the samples. For the XRD spectrum of the synthesized 100 nm and 20 µm crystals, the diffraction peaks at 2θ of 6.75°, 9.5°, 9.7°,13.5°, and 19.1° correspond to HKUST-1 single-crystal diffraction peaks in the simulation results, respectively, revealing the complete crystal structure of 100 nm and 20 µm HKUST-1.

### 2.2. Iodine Adsorption Kinetics

**Figure 4:**
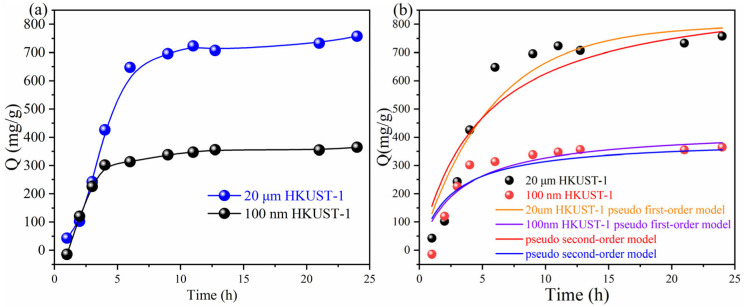
(**a**) Effect of adsorption time on the capacity at 80 °C; (**b**) pseudo-first-order model and the pseudo-second-order model fitting curve.

Figure 4a shows the adsorption kinetics curves of two different particle sizes of HKUST-1 (100 nm and 20 μm) under a saturated iodine vapor environment at 80 °C. For 100 nm HKUST-1, adsorption equilibrium is reached in approximately 5 h. For 20 μm HKUST-1, adsorption equilibrium is achieved after about 8 h. This difference is likely due to the smaller particle size of the 100 nm HKUST-1, which has a larger specific surface area and more accessible surface pores, allowing iodine molecules to diffuse into the pores more rapidly and leading to a faster adsorption rate. In contrast, the 20 μm HKUST-1, with larger crystal sizes, requires a longer diffusion path for iodine molecules, resulting in a slower adsorption process. For 100 nm HKUST-1, saturated adsorption capacity is approximately 300 mg/g. For 20 μm HKUST-1, saturated adsorption capacity is around 800 mg/g, which is significantly higher than the 100 nm sample. This can be attributed to the fact that iodine adsorption is mainly influenced by the internal pore structure of HKUST-1. The 20 μm HKUST-1 crystals, with higher crystallinity and a more complete pore network, provide more internal space for iodine molecule adsorption, which is the primary reason for their higher adsorption capacity compared to the 100 nm HKUST-1 crystals. Additionally, the larger crystal structure is more stable, reducing the risk of pore collapse and further enhancing the adsorption capacity.

Figure 4b shows the kinetic fitting curves of iodine adsorption on HKUST-1 particles at 80 °C, utilizing both the pseudo-first-order and pseudo-second-order models. It can be observed that the pseudo-second-order model provides a better fit to the experimental data for both 20 µm and 100 nm HKUST-1 particles, as indicated by the closer alignment of the model curves to the data points. This suggests that the adsorption process is likely governed by chemisorption, which involves valence forces through the sharing or exchange of electrons. Furthermore, the adsorption capacity of the 20 µm HKUST-1 is significantly higher than that of the 100 nm HKUST-1 across all time points, highlighting the impact of particle size on the adsorption performance. The faster initial adsorption rate for the 20 µm HKUST-1 compared to the 100 nm HKUST-1 is also evident, likely due to differences in the diffusion pathways and surface availability of iodine molecules.

The adsorption kinetics curve of HKUST-1 for iodine was linearly fitted using the following two models [25,26]:

The pseudo-first-order kinetic model, as shown in Equation (1):

(1) $$\ln\left(q_{e}-q_{t}\right)=lnq_{e}-k_{1st}t$$

The pseudo-second-order kinetic model, as shown in Equation (2):

(2) $$(\frac{t}{q_{t}})=\frac{1}{k_{2nd}q_{e}^{2}}+(\frac{1}{q_{e}})t$$

where *q_t_* is the adsorption capacity at time *t*, *q_e_* is the equilibrium adsorption capacity of the resin, and *k*_1*st*_ and *k*_2*nd*_ are the rate constants for the pseudo-first-order kinetics and pseudo-second-order kinetics, respectively. The fitting results are presented in Table 1.

Figure 4b and Table 1 show the equation fitting curves of 20 µm and 100 nm HKUST-1 iodine adsorption curves, as well as the fitting results of related parameters. Although the pseudo-first-order model shows a relatively high *R*^2^, there is a noticeable discrepancy between the calculated *q_e_* values and the experimental values, indicating that the pseudo-first-order kinetic model only provides a general fit for iodine adsorption by HKUST-1. In contrast, the pseudo-second-order kinetic model, with an *R*^2^ value close to 1, demonstrates a much better description of the iodine adsorption process. The fitted *q_e_* values are in close agreement with the experimental data, further supporting the conclusion that HKUST-1’s adsorption behavior aligns more closely with the pseudo-second-order model.

Based on the pseudo-second-order kinetic model fitting results, the iodine adsorption process by HKUST-1 is primarily physical adsorption. Physical adsorption is typically associated with higher porosity and surface area, which aligns with the three-dimensional open-pore structure of HKUST-1.

**Figure 5:**
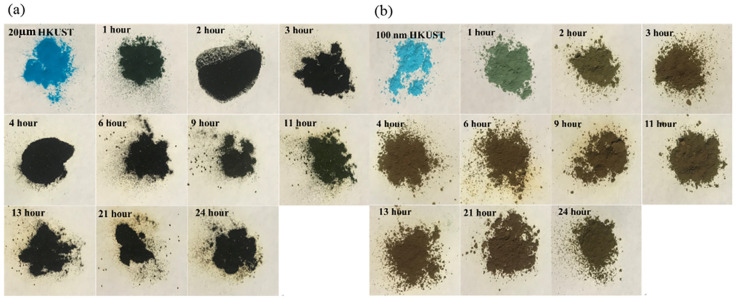
Images of (**a**) 20 μm and (**b**) 100 nm HKUST-1 at different times during iodine adsorption at 80 °C (**a**) 20 μm HKUST-1 (**b**) 100 nm HKUST-1.

Figure 5 shows the color changes of 20 μm and 100 nm HKUST-1 materials after iodine adsorption at 80 °C over different time periods. The gradual darkening of the samples visually reflects the iodine adsorption capacity and the dynamic process. Both sizes of HKUST-1 initially display a blue color, characteristic of Cu(II) ions in the HKUST-1 material, indicating no iodine adsorption. For 20 μm HKUST-1, the color changes rapidly from blue to dark green after one hour, and then gradually darkens to brown/black. As the adsorption time increases (13–24 h), the color deepens further, indicating that iodine molecules are continuously being adsorbed into the internal pores of HKUST-1. The darkening color is consistent with the high adsorption capacity, confirming that 20 μm HKUST-1 has a stronger iodine adsorption ability. In contrast, 100 nm HKUST-1 shows a faster color change, but the final color is lighter, displaying only a brownish hue and not reaching the depth observed in the 20 μm sample. This suggests that, while 100 nm HKUST-1 adsorbs iodine rapidly in the initial stages, its overall adsorption capacity is limited, with less iodine adsorbed when equilibrium is reached. This result is in agreement with the adsorption kinetics curve in Figure 4, where the adsorption capacity of 20 μm HKUST-1 reaches around 700–800 mg/g, indicating higher adsorption performance, while 100 nm HKUST-1 only achieves an adsorption capacity of 300–400 mg/g, reflecting lower efficiency.

The higher iodine adsorption capacity of 20 μm HKUST-1 is due to its larger crystal size, which results in higher crystallinity and a more complete three-dimensional pore structure. This provides more internal pores for iodine molecules to diffuse and adsorb. The lower diffusion resistance within the pores helps iodine molecules penetrate deeper into the crystal, thereby enhancing the adsorption capacity. In contrast, despite its faster adsorption rate, the lower capacity of 100 nm HKUST-1 is due to its smaller particle size and greater number of external surface pores. Initially, iodine molecules are more easily adsorbed on the external surface, leading to a faster adsorption rate. However, the limited number of internal pores and smaller adsorption space within the crystal result in a lower final saturation capacity compared to 20 μm HKUST-1.

### 2.3. Instrumental Characterization After Adsorption

**Figure 6:**
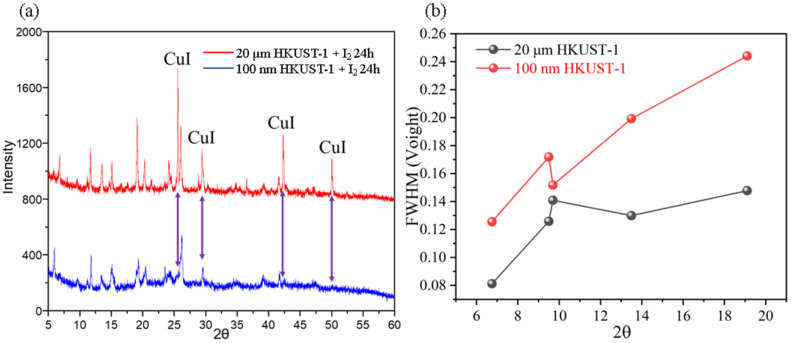
(**a**) XRD patterns of 100 nm and 20 μm HKUST-1 after 24 h of iodine adsorption; (**b**) comparison of half-width values of characteristic peaks at 20 um and 100 nm HKUST-1.

The XRD characterization of HKUST-1 after iodine adsorption is shown in Figure 6a. Compared to the XRD pattern of HKUST-1 before adsorption, significant changes in both peak positions and intensities are observed after iodine adsorption. Specifically, the XRD patterns of 20 μm HKUST-1 and 100 nm HKUST-1 after iodine adsorption differ in peak positions and intensities, suggesting that different amounts of iodine molecules have filled the internal pores of HKUST-1 with varying particle sizes, leading to distinct diffraction peaks.

Figure 6a presents the XRD patterns of 100 nm and 20 μm HKUST-1 after 24 h of iodine adsorption at 80 °C. Comparing these with the XRD patterns of the samples before adsorption, it is evident that both the peak positions and intensities have changed significantly, indicating that the iodine molecules entering the HKUST-1 pores have affected the crystal structure. After iodine adsorption, the XRD patterns of HKUST-1 still display the characteristic diffraction peaks of the original HKUST-1; however, some peaks show slight shifts, suggesting that the introduction of iodine molecules has altered the lattice parameters of the HKUST-1 framework. As iodine molecules enter the pores of HKUST-1, they interact with copper clusters or other local structures within the framework, leading to microstructural changes.

The diffraction peaks of 20 μm HKUST-1 (red line) in Figure 6a are sharper and more intense, indicating that it retains higher crystallinity after iodine adsorption. Similarly, Figure 6b shows that the half-width values of the five characteristic peaks for 20 μm HKUST-1 are consistently lower than those of 100 nm HKUST-1, further confirming its superior crystallinity. This is attributed to the higher initial crystallinity and structural stability of the larger 20 μm crystals. In contrast, the diffraction peaks of 100 nm HKUST-1 (blue line) exhibit significantly lower intensity and broader shapes, suggesting substantial disruption to its crystal structure during iodine adsorption, leading to a pronounced reduction in local crystallinity. The better framework stability and more complete pore structures of the larger 20 μm HKUST-1 crystals allow them to maintain their crystallinity with minimal changes to the diffraction pattern. On the other hand, the smaller 100 nm HKUST-1 crystals, due to their higher sensitivity to iodine molecule filling, experience partial pore deformation or collapse, resulting in decreased peak intensity and broader diffraction peaks.

The XRD analysis reveals that the iodine molecules have different effects on the crystal frameworks of HKUST-1 depending on the particle size. For 20 μm HKUST-1, iodine molecules predominantly fill the internal pores without significantly altering the framework structure, as evidenced by the high intensity and sharpness of the diffraction peaks. This behavior is linked to its larger pore volume and higher crystallinity, which allow it to accommodate more iodine molecules while maintaining structural integrity. For 100 nm HKUST-1, the iodine filling causes local structural disturbances, with some pore structures undergoing slight deformation, leading to decreased peak intensity. Given the limited number of pores in nanoscale HKUST-1, the framework structure is more sensitive to iodine molecule filling.

The XRD results suggest that the introduction of iodine molecules primarily involves a physical filling process, where iodine enters the three-dimensional pores of HKUST-1, forming stable guest–host interactions. HKUST-1 materials of different particle sizes exhibit varying adsorption capacities and structural stabilities. With its stable structure, higher iodine adsorption capacity, and retained diffraction peak intensity, 20 μm HKUST-1 shows superior performance. In contrast, 100 nm HKUST-1 experiences local structural disturbances, and the increased peak width suggests a slight reduction in the microscopic stability of the framework.

**Figure 7:**
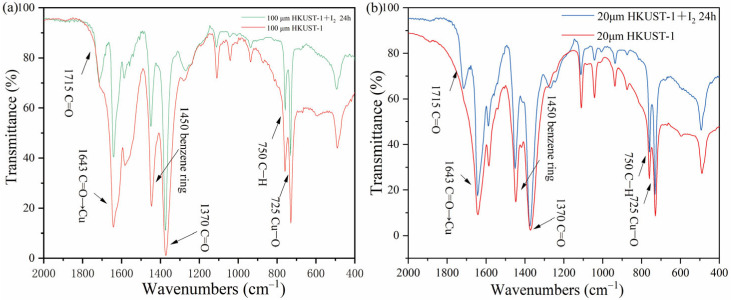
FTIR spectra of (**a**) 100 nm and (**b**) 20 μm HKUST-1 after iodine adsorption.

Figure 7a,b show the Fourier transform infrared (FTIR) spectra of 100 nm and 20 μm HKUST-1 before and after iodine adsorption. By comparing the changes in transmittance within the wavenumber range, the interaction between iodine molecules and the HKUST-1 material, as well as the adsorption mechanism, can be revealed.

From Figure 7a, it can be seen that the FTIR spectrum of 100 nm HKUST-1 before iodine adsorption (red line) shows a characteristic C=O stretching vibration peak around 1700 cm^−1^, which is attributed to the benzene-1,3,5-tricarboxylate (BTC) ligand in the HKUST-1 material. A C–H out-of-plane bending vibration peak is observed in the 700–900 cm^−1^ range, indicating the characteristic vibrations of the benzene ring. Peaks in the 400–600 cm^−1^ range correspond to Cu–O bond vibrations, which are characteristic of the copper clusters and ligands within the HKUST-1 framework.

After iodine adsorption, the FTIR spectrum of 100 nm HKUST-1 (green line) shows a weakening of the characteristic peaks, particularly in the 700–900 cm^−1^ and 400–600 cm^−1^ regions. New vibration peaks appear, with a characteristic peak near 500 cm^−1^, which reflects the physical adsorption and weak interactions between iodine molecules and the copper clusters or the inner walls of the pores in the framework. The C=O stretching vibration peak shifts slightly, indicating that iodine molecules filling the pores within the framework affect the structure of the BTC ligands.

In Figure 7b, for 20 μm HKUST-1 before iodine adsorption (red line), similar to the 100 nm sample, the C=O stretching vibration peak near 1700 cm^−1^ and the C–H bending vibration peak of the benzene ring in the 700–900 cm^−1^ region are clearly visible. The Cu–O vibration peak is also observed in the 400–600 cm^−1^ range, indicating that the framework structure is intact. After iodine adsorption, the FTIR spectrum of 20 μm HKUST-1 (blue line) shows more noticeable changes in peak intensities, especially in the 400–600 cm^−1^ range corresponding to the Cu–O bonds. This suggests that iodine molecules entering the pores exert a stronger impact on the surrounding structure of the copper clusters. A characteristic peak near 500 cm^−1^ appears, similar to the 100 nm HKUST-1, further confirming the physical adsorption and weak interactions between iodine molecules and the framework pores. Compared to the 100 nm HKUST-1, the changes in the FTIR peak intensities of the 20 μm HKUST-1 are more significant, indicating that it adsorbed more iodine molecules, leading to a more pronounced effect on the structural vibrations.

By comparing the two figures, it is evident that both samples exhibit a new characteristic peak around 500 cm^−1^ after iodine adsorption, confirming that iodine molecules have filled the pores and undergone physical adsorption with the framework. A slight shift in the C=O vibration peak indicates that the framework structure is affected by the iodine molecule filling. The main difference is that for 20 μm HKUST-1, the changes in peak intensities are more pronounced, particularly in the Cu–O region, suggesting it adsorbed more iodine molecules. In contrast, the 100 nm HKUST-1 shows smaller structural disturbances, reflecting its lower adsorption capacity and less significant impact on the framework structure.

### 2.4. Adsorption Stability of HKUST-1

**Figure 8:**
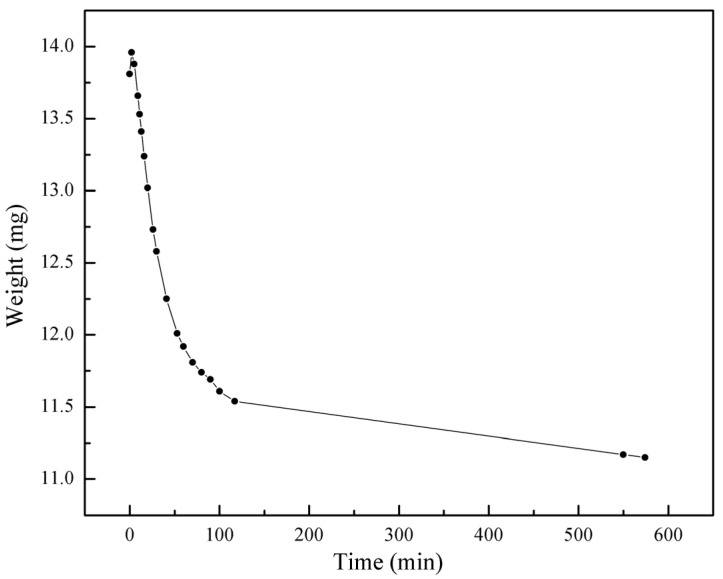
Weight changes of 20 μm HKUST-1 after iodine adsorption when exposed to air.

Figure 8 illustrates the weight change of 20 μm HKUST-1 after iodine adsorption when exposed to air over time. As shown in the graph, the sample’s weight gradually decreases over time, reflecting the volatilization behavior of adsorbed iodine and the ability of the HKUST-1 framework to retain iodine molecules.

In the initial phase (0–100 min), the weight decreases rapidly from 14.0 mg to approximately 11.5 mg, indicating that a large amount of iodine is quickly released from the surface and volatile pores of HKUST-1. This stage corresponds to the rapid desorption of iodine from the surface and some of the iodine within the pores, representing a fast desorption process. Since iodine molecules are primarily physically adsorbed within the HKUST-1 pores, some molecules tend to volatilize when exposed to air.

In the subsequent phase (100–600 min), after 100 min, the weight change slows down, with the sample weight gradually decreasing from 11.5 mg to around 11.0 mg. This phase reflects the slower volatilization of iodine molecules from deeper pores, where longer diffusion paths reduce the volatilization rate. At this point, the HKUST-1 framework’s ability to retain iodine becomes more apparent, as stronger physical constraints limit the rapid release of iodine.

The weight change can be divided into two distinct stages: the rapid desorption stage, where iodine molecules on the surface volatilize quickly due to their high volatility and weak physical adsorption; and the slow desorption stage, where iodine volatilization from deeper pores is hindered by diffusion resistance and physical constraints from the framework. The more complete three-dimensional pore structure of 20 μm HKUST-1 allows for deeper iodine filling, leading to a gradual decrease in the desorption rate.

The overall trend indicates that, despite gradual volatilization, 20 μm HKUST-1 retains a significant amount of iodine, particularly in the later stages, where the weight loss slows. This is attributed to the high crystallinity and well-developed pore structure of 20 μm HKUST-1. The framework’s high crystallinity enhances its physical constraints on iodine, while the larger pore network offers greater capacity for iodine retention, slowing down the volatilization process.

**Figure 9:**
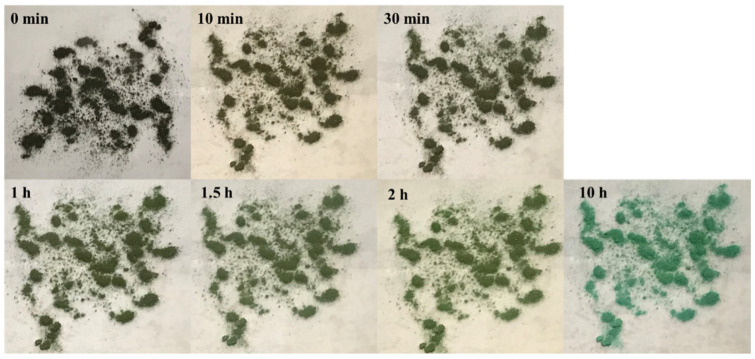
Photos of 20 μm HKUST-1 exposed to air for 0 min, 10 min, 30 min, 1 h, 1.5 h, 2 h, and 10 h.

Figure 9 presents the color changes of 20 μm HKUST-1 after iodine adsorption at various time intervals (0 min, 10 min, 30 min, 1 h, 1.5 h, 2 h, and 10 h) when exposed to air. It is clearly evident that as the exposure time increases, the sample’s color gradually transitions from black to green, eventually returning to the original color of the HKUST-1 before iodine adsorption. This phenomenon is consistent with the gradual weight loss observed in Figure 8, reflecting the desorption behavior of the iodine molecules and the ability of the HKUST-1 framework to retain iodine.

At 0 min, the iodine-loaded 20 μm HKUST-1 appears deep black, indicating that a significant amount of iodine molecules has filled the pores and surface. As the exposure time increases (from 10 min to 2 h), the sample color gradually lightens to light brown, suggesting that some iodine molecules have desorbed from the surface and shallow pores, causing the color to fade. After 10 h, the sample’s color nearly returns to the original green of HKUST-1, indicating that most of the physically adsorbed iodine molecules have volatilized from the pores. This process illustrates the physical adsorption nature of iodine, where the interaction between iodine molecules and the HKUST-1 framework is relatively weak, making iodine readily desorb when exposed to air.

The color change pattern indicates that iodine desorption primarily occurs at the surface and shallow pores of HKUST-1. This is because iodine molecules in these regions experience less diffusion resistance and are more easily volatilized from the material. In contrast, iodine molecules in deeper pores are restricted by longer diffusion paths, resulting in slower desorption. Therefore, it takes longer for iodine in these deeper pores to be completely released.

Combining the weight loss data from Figure 8, after 10 h of exposure to air, the weight of the 20 μm HKUST-1 decreases by about 20%. This weight loss aligns with the observed color changes, further confirming the occurrence of the desorption process. In the initial rapid desorption phase (0–1 h), the weight drops quickly, and the color change is pronounced. This stage is primarily characterized by the rapid volatilization of iodine from the surface and shallow pores, leading to a significant decrease in sample weight. In the subsequent slow desorption phase (1–10 h), the weight loss becomes slower, and the color stabilizes, with the sample approaching its initial state before iodine adsorption. The desorption in this phase is mainly controlled by the slow diffusion and release of iodine molecules from the deeper pores. Due to the higher pore integrity and structural stability of 20 μm HKUST-1, iodine molecules are more physically constrained in the deeper pores, resulting in a significantly slower desorption rate.

## 3. Synthesis of Adsorbents and Experimental Methods

### 3.1. Synthesis of HKUST-1

For 100 nm HKUST-1 [27], 1.0 mmol of benzene-1,3,5-tricarboxylic acid, >99%) was dissolved in a mixture of 6 mL deionized water and 6 mL ethanol. In a separate beaker, 1.5 mmol of copper nitrate trihydrate (Cu(NO_3_)_2_•3H_2_O, Tianjin, China, Guangfu, analytical grade) and 3 mmol of sodium formate (Kaiyuan, China, Shenxin, >99%) were dissolved in a mixture of 6 mL deionized water and 6 mL ethanol. The two solutions were then mixed under magnetic stirring (T = Room Temperature, R= 120 r/min) and transferred to a 100 mL hydrothermal reaction vessel, where the reaction was carried out at 120 °C for 24 h. After the reaction, the mixture was centrifuged and washed three times with water and ethanol, then dried overnight at room temperature.

For 20 μm HKUST-1 [28], 1.0 g of benzene-1,3,5-tricarboxylic acid was dissolved in a mixture of 30 mL ethanol (Tianjin, China, Kemiou, analytical grade) and DMF (Shanghai, China, Aladdin, >98%) in a 1:1 volume ratio. In a separate beaker, 2.804 g of copper nitrate trihydrate (Cu(NO_3_)_2_•3H_2_O, Shanghai, China, Macklin, AR) was dissolved in 15 mL of deionized water. The two solutions were mixed and stirred at room temperature for 10 min, then transferred to a hydrothermal reaction vessel and reacted at 100 °C for 10 h. The product was filtered, washed three times with ethanol, and dried at room temperature.

### 3.2. Iodine Adsorption

A total of 20 mg of 100 nm and 20 μm HKUST-1 adsorbents were placed in three 2 mL small screw-cap weighing bottles, and the initial weights of the bottles were recorded. Excess iodine particles (analytical grade, Aladdin) were added to one 5 mL screw-cap weighing bottle. The four weighing bottles were then grouped together in a larger 70 mm × 40 mm weighing bottle. Ten sets of weighing bottles were prepared and placed in an oven at 80 °C for adsorption kinetics testing. At fixed time intervals, one set of bottles was removed, and the small bottles were taken out. They were placed in an oven for 1 min to remove gaseous iodine, after which the bottles were capped and weighed. The weighing bottles were not returned to the oven after weighing.

### 3.3. Characterization

The morphology of the synthesized adsorbent crystals was examined using a scanning electron microscope (SEM, Tokyo, Japan, Hitachi, Model S-4800). The samples were mounted on conductive adhesive tape and pre-treated by gold sputtering, with an electron beam acceleration voltage of 20 kV. The crystal structure was characterized by X-ray diffraction (XRD) using a Bruker AXS GmbH diffractometer (Billerica, MA, USA, Model D8-Focus, Cu target). Powdered samples were analyzed using the powder diffraction method, with a scanning range of 4–50°, a scan rate of 10°/min, and a step size of 0.01°. The functional group structure of iodine-loaded HKUST-1 was analyzed using Fourier transform infrared spectroscopy (FTIR, Tianjin, China Tianjin Port East FTIR-650). KBr pellets were used for testing, with a scanning range of 4000 to 400 cm^−1^.

## 4. Conclusions

This study systematically investigates the iodine vapor adsorption performance of two different particle sizes of the metal–organic framework material HKUST-1 (100 nm and 20 μm), with a detailed analysis of its structural stability and iodine desorption behavior. The main conclusions are as follows:

1. HKUST-1 materials with particle sizes of 100 nm and 20 μm were successfully synthesized. The 20 μm HKUST-1 exhibits higher crystallinity and a more complete pore structure. In an 80 °C iodine vapor environment, the saturated iodine adsorption capacity of 20 μm HKUST-1 reaches 700 mg/g, significantly higher than the 300 mg/g of the 100 nm sample.
2. The iodine adsorption behavior of HKUST-1 follows a pseudo-second-order kinetic model, primarily driven by physical adsorption, with iodine molecules filling the pores without disrupting the overall framework structure. The 20 μm HKUST-1 demonstrates higher internal diffusion rates and superior structural stability.
3. After iodine adsorption, the 20 μm HKUST-1 shows a self-desorption phenomenon when exposed to air, with its weight decreasing by approximately 20% within 10 h and its color gradually returning to green. To improve long-term iodine retention, future studies may focus on modifying the material to enhance its binding stability.

## Figures and Tables

**Table 1:** Kinetic fitting parameters for HKUST-1. (The saturated vapor pressure of the iodine at 80 °C.)

Particle Size	Pseudo-First-Order Dynamics Model			Pseudo-Second-Order Dynamics Model		
	*k*_1*st*_	*q_e_*	*R* ^2^	*k*_2*nd*_	*q_e_*	*R* ^2^
	(1/h)	(mg/g)	/	(g/mg/h)	(mg/g)	/
100 nm	0.293	356.45	0.953	1.83 × 10^−3^	384.62	0.997
20 μm	0.325	1125.1	0.975	1.06 × 10^−3^	769.23	0.998

## Data Availability

Data are contained within the article.
